# Mitochondrial genomes as living ‘fossils’

**DOI:** 10.1186/1741-7007-11-30

**Published:** 2013-04-15

**Authors:** Ian Small

**Affiliations:** 1 Australian Research Council Centre of Excellence in Plant Energy Biology, Bayliss Building, The University of Western Australia, 35 Stirling Highway, Crawley, WA 6009, Australia

## Abstract

The huge variation between mitochondrial genomes makes untangling their evolutionary histories difficult. Richardson *et al.* report on the remarkably unaltered ‘fossil’ genome of the tulip tree, giving us many clues as to how the mitochondrial genomes of flowering plants have evolved over the last 150 million years, and raising questions about how such extraordinary sequence conservation can be maintained.

See research article http://www.biomedcentral.com/1741-7007/11/29.

## Commentary

Although they are all thought to derive from a single endosymbiotic relationship, mitochondrial genomes show extraordinary diversity. They include examples of the smallest (non-viral) genomes known and examples larger than most bacterial genomes [1]. They include some of the fastest mutating genomes and some of the slowest [1]. In metazoans, mitochondrial gene order is usually invariant over tens or even hundreds of millions of years, whereas in plants, mitochondrial gene order often differs between organelles in the same cell [2]. Some use different genetic codes and some alter the sequence of their RNA to code for proteins that are not coded in the genome [3]. Together, they offer fascinating insights into evolutionary processes at work. The difficulty lies in extrapolating backwards from present day diversity to imagine what the ancestral state at any one point might have been. The new paper by Richardson *et al.*[4] not only provides yet another record-breaking mitochondrial genome with its share of ‘gee-whizz’ characteristics, it also turns out to be an extremely useful window into the past.

**Figure 1.**
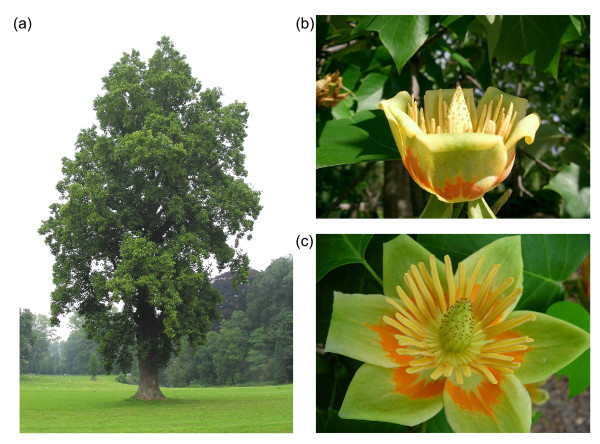
**Tulip trees are a popular feature in parks and gardens. (a) **The American tulip tree, *Liriodendron tulipifera*. Photo by Jean-Pol Grandmont. **(b) **Side view of a flower, the shape of which gives the tree its name. **(c) **Top view of a flower.

Richardson *et al*. chose to study the tulip tree (*Liriodendron tulipifera* L.), a native of the eastern United States, but widely grown elsewhere as a magnificent specimen tree in parks and gardens (Figure 1a). The spectacular flowers (Figure 1b, c) make it clear that this is a flowering plant (Magnoliophyta), but most admirers won’t realize that the tulip tree is a member of an early-branching lineage, quite separate from the bulk of flowering plants in the monocot and dicot clades. As all our important crop plants lie in these two dominant clades, they have been the targets of almost all the sequencing efforts around the world. As a result, for many of the important molecular traits that differ between monocots and dicots, we cannot tell which is the ancestral state. The new sequence from the tulip tree helps to understand what genes were present in the ancestral flowering plant mitochondrial genome, which ones were clustered together, which ones were captured from chloroplast DNA and when, which ones contained introns, and the degree to which each gene required editing of its RNA transcripts. It answers these questions particularly convincingly because of its most startling characteristic - the mitochondrial genome of the tulip tree (and those of its relatives, the magnolias) contain the most slowly evolving genes ever studied. Some of the *Magnolia* genes appear to have acquired no mutations at all in 50 million years [4]. This ‘fossilized’ genome gives us some important clues as to what mitochondrial genomes looked like (and how they functioned) as flowering plants evolved and took over the land in the time of the dinosaurs.

### ‘Fossilizing’ a genome

Like all good science, this work raises as many questions as it answers, and one obvious one that will exercise the minds of researchers everywhere is how can an organism so effectively prevent its genome from acquiring mutations? Mitochondrial genomes are subject to recurrent damage from reactive oxygen species generated by the respiratory electron transport chain, not to mention unavoidable polymerase errors during DNA replication [5]. Mitochondria are generally uniparentally inherited in plants, limiting the possibility of eliminating mutations by sexual recombination, and, in any case, plant mitochondrial genomes show very low rate of change at silent sites too, not just functionally important sites. Hence, low mutation rates are largely due to mechanisms that suppress or correct mutations, rather than to natural selection.

One factor of uncertain importance is life span. Genomes of long-lived organisms tend to be copied less often, but it is unclear how important this is in plants, which lack a clearly segregated germ line, and can reproduce at a relatively young age, even in long-lived species. Probably of more importance is that long-lived organisms tend to have much stronger defenses against genotoxic damage, due to selection against premature somatic senescence [6]. Indeed, there is very active research into the possible links between mitochondrial genome damage and aging in humans [5]. The tulip tree comes from a long line of woody perennials with relatively long life spans and can live to 300 to 400 years old (the oldest living organism in New York is reputed to be a tulip tree, the ‘Queens Giant’). It is likely, therefore, that it possesses excellent biochemical protection (antioxidants and the like) against genome damage.

Plant mitochondria also possess highly active mismatch repair and recombination machinery. Recent advances by several labs have identified many of the components of this machinery, some of which are clearly derived from the original bacterial endosymbiont, some of which appear to be novel. Key players include the DNA mismatch repair protein MSH1 (related to bacterial MutS) and a small family of RecA homologs likely to be involved in DNA strand exchange during homologous recombination [7]. Active strand exchange and gene conversion amongst the multiple copies of the mitochondrial genome in a single cell are likely to act to suppress the fixation of mutations [2], but as yet there have not been any comparisons between the components active in species with fast-mutating genomes (for example, some *Silene*[1]) or slow-mutating genomes (for example, *Liriodendron*). As the rate of fixation of mutations can vary by 5,000-fold [4], we might expect some fairly obvious differences to be revealed were such a comparison to be made.

### ‘Fossilizing’ a proteome

Plant organelles (both mitochondria and chloroplasts) have a third line of defense in addition to biochemical protection from genome damage and post-damage repair of DNA lesions. They can edit their RNA to alter its coding function, enabling proteins to be produced that were ‘incorrectly’ coded in the DNA sequence. RNA editing depends on large numbers of highly specific trans-factors, each of which is capable of targeting a specific base to be edited (reviewed in [3]). In general, RNA editing restores conserved amino acids and leads to the protein sequences resembling the ancestral sequence more closely than the codon sequence in the DNA does. In this way, the proteome of plant organelles is more highly conserved than the genome. The extent to which this process occurs varies widely, with the highest rates of RNA editing seen in early diverging vascular plants such as ferns and lycophytes and the lowest seen in very early diverging lineages such as some liverworts [8]. Flowering plants have intermediate levels of RNA editing (for example, approximately 500 sites in *Arabidopsis* organelles). This has led to proposals that following rapid accumulation of new editing sites during the evolution of land plants, flowering plants may now be losing editing sites through random back-mutation and reverse transcription of edited transcripts (reviewed in [3]).

Richardson *et al*. provide important new data on the evolution of RNA editing in plants. The *Liriodendron* mitochondrial transcriptome is edited at at least 781 sites, more than in any other flowering plant studied to date [4]. Many of these sites are at identical positions to those found in monocots, dicots or both, indicating that they are ancestral sites, not newly created sites. This is strong evidence that the earlier proposals are correct (flowering plants are tending to lose more sites than they gain). What could be the drivers for these long-term tendencies in creation and loss of RNA editing sites? Overall, there is a positive correlation between C-to-U RNA editing and GC content of the genome [9], so it is likely that it is mutation/repair processes at the genome level that are driving changes in editing frequency over evolutionary timescales. One highly speculative theory is that changes in UV damage (and thus rate of genomic mutation of T to C) may be the primary cause of these trends, but it could equally be inherent biases during gene conversion events [9].

### The future is in the past

The increasing cost-effectiveness of genome and transcriptome sequencing is making it easier for researchers to strategically choose the most informative species rather than having to make do with those that are economically important and therefore easier targets for funding. The coverage of early diverging plants is still far from optimal, with many large and important groups still barely sampled (for example, gymnosperms and ferns). Ideally, we would like all three genomes at once, as a comparison of the two organelle genomes is often highly informative, and the nuclear genome provides the sequences of all of the potential molecular factors influencing the processes under study. In this context, a particularly exciting development is the elucidation of the molecular basis for sequence recognition by RNA editing factors [10], permitting prediction of which factor edits which RNA site [11]. This breakthrough will greatly accelerate comparative genomics of RNA editing and adds lots of value to any complete genome sequence. I look forward to being able to analyze the next molecular ‘fossil’ to roll off the sequencing machines!
